# DIgital Alcohol Management ON Demand (DIAMOND) feasibility randomised controlled trial of a web-based intervention to reduce alcohol consumption in people with hazardous and harmful use versus a face-to-face intervention: protocol

**DOI:** 10.1186/s40814-015-0023-1

**Published:** 2015-08-08

**Authors:** Fiona L. Hamilton, Jo Hornby, Jessica Sheringham, Sally Kerry, Stuart Linke, Francesca Solmi, Charlotte Ashton, Kevin Moore, Elizabeth Murray

**Affiliations:** 1 eHealth Unit, Department of Primary Care & Population Health, University College London, London, UK; 2 Department of Applied Health Research, University College London, London, UK; 3 Centre for Primary Care and Public Health, Queen Mary University, London, UK; 4 Camden and Islington NHS Foundation Trust, London, UK; 5 Camden and Islington Public Health, London, UK; 6 Royal Free Hospital, London, UK

**Keywords:** Alcohol, Brief intervention, Digital intervention, Randomised controlled trial

## Abstract

**Background:**

“Hazardous and harmful” drinkers make up approximately 23 % of the adult population in England. However, only around 10 % of these people access specialist care, such as face-to-face extended brief treatment in community alcohol services. This may be due to stigma, difficulty accessing services during working hours, a shortage of trained counsellors and limited provision of services in many places. Web-based alcohol treatment programmes may overcome these barriers and may better suit people who are reluctant or unable to attend face-to-face services, but there is a gap in the evidence base for the acceptability, effectiveness and cost-effectiveness of these programmes compared with treatment as usual (TAU) in community alcohol services.

This study aims investigate the feasibility of all parts of a randomised controlled trial (RCT) of a psychologically informed web-based alcohol treatment programme called Healthy Living for People who use Alcohol (HeLP-Alcohol) versus TAU in community alcohol services, e.g. recruitment and retention, online data collection methods, and the use and acceptability of the intervention to participants.

**Methods:**

A feasibility RCT delivered in north London community alcohol services, comparing HeLP-Alcohol with TAU. Potential participants are aged ≥18 years referred or self-referred for hazardous and harmful use of alcohol, without co-morbidities or other complex problems. The main purpose of this study is to demonstrate the feasibility of recruiting participants to the study and will test online methods for collecting baseline demographic and outcome questionnaire data, randomising participants and collecting 3-month follow-up data. The acceptability of this intervention will be measured by recruitment and retention rates, automated log-in data collection and an online service satisfaction questionnaire. The feasibility of using tailored text message, email or phone prompt to maintain engagement with the intervention will also be explored. Results of the study will inform a definitive Phase 3 RCT.

**Results:**

Recruitment started on 26 September 2014 and will run for 1 year.

**Conclusion:**

The proposed trial will provide data to inform a fully powered non-inferiority effectiveness and cost-effectiveness RCT comparing HeLP-Alcohol with TAU.

**Trial registration:**

ISRCTN31789096.

## Background

Alcohol is the second biggest lifestyle risk factor for disease and premature mortality after smoking [1]. Alcohol misuse is estimated to cost the UK around £21 billion per year [2, 3]. This includes National Health Service (NHS) costs for treating accidents, assaults resulting from alcohol intoxication, conditions such as liver disease, cardiovascular disease and cancers, and the cost of alcohol treatment services. Societal costs include those associated with absenteeism, unemployment, family breakdown, road traffic collisions and crime.

These costs are mainly due to the large number of “hazardous and harmful” drinkers who make up approximately 23 % of the adult population; about 7.1 million people in England [4]. Hazardous drinkers are those whose alcohol intake is greater than government recommended limits (14 units a week for women and 21 units for men; or 2–3 units daily for women and 3–4 units for men). Harmful drinkers are those drinking more than recommended limits, and experiencing alcohol-related harm, but without symptoms of physical or psychological dependence. About 3.6 % of the population (1.1 million) people in England are dependent on alcohol.

People drinking at hazardous or harmful levels can respond well to a tiered approach to treatment [5]. Tier 1 is identification and brief advice (IBA), also known as screening and brief intervention (SBI). This may be conducted by a health professional in any setting, for example general practice or accident and emergency department (A&E). Identification is through the use of validated questionnaires such as the Alcohol Use Disorders Identification Test (AUDIT) [6], or shortened versions of AUDIT such as AUDIT Consumption (AUDIT-C) [7] or the Fast Alcohol Screening Test (FAST) [8]. Brief advice involves a short conversation about the risks of alcohol use at the current level, advice on reducing drinking levels and sources of self-help, usually supplemented with a leaflet [9]. Tier 2/3 treatments are provided at community alcohol drug and alcohol services, which offer more specialised treatment for people who need more than IBA, including extended brief intervention face-to-face, either individually or in groups, and complimentary therapies tailored to the individual. Tier 4 is detoxification and other treatment targeted at dependent drinkers with significant co-morbidities such as liver disease, severe mental health problems or multiple substance misuse.

IBA for hazardous and harmful drinking results in a significant and sustained reduction in alcohol consumption, which has been quantified in a meta-analysis by Kaner et al. [10] as a mean difference of −38 g of alcohol a week (CI −53 to −23 g) compared with control treatments. This translates to a reduction in average weekly intake of around four standard UK drinks (1 unit = 8 g alcohol). IBA is also likely to be cost-saving [11]. Despite this, not everyone who drinks at hazardous or harmful levels will find IBA sufficient to help them reduce their drinking and will need to access Tier 2/3 services.

There are enormous challenges in meeting demand for Tier 2/3 services, and estimates suggest only 6 to 10 % of people with problem alcohol use are accessing specialist treatment in England [4, 12]. Over the last several years commissioners of drug and alcohol services have faced a funding deficit [13]. This has inevitably resulted in a shortage of trained alcohol counsellors, with skills over and above the generic competencies that all primary care and A&E health care professionals need to provide IBA. In addition, hazardous or harmful alcohol use is recognised to be a relapsing and remitting condition so patients may need to access help repeatedly. Even if services are available, people may be reluctant to attend due to perceived stigma of having alcohol problems, or they may work and so cannot attend during working hours.

Online interventions could address these challenges. Internet use is widespread: 84 % of households in Great Britain having access to the internet, 76 % of adults use the internet every day and 58 % use a mobile phone to do so [14]. Online interventions are relatively inexpensive to set up, are convenient to access and provide anonymity, which is important to people with stigmatising conditions. A recent systematic review has shown that online interventions may be effective and cost-effective in treating hazardous and harmful alcohol use [15]. The feasibility of providing online interventions similar to Tier 2/3 community alcohol services has been demonstrated and also found to be acceptable to alcohol counsellors [16]. However, much of the research to date has looked at online interventions for IBA, largely in student populations. There are relatively few data demonstrating the effectiveness of extended brief intervention in adult populations of hazardous and harmful drinkers, and no data comparing face-to-face treatment with online treatment.

A fully powered Phase 3 randomised controlled trial (RCT) could address these gaps in knowledge, but there are recognised challenges with trials of web-based interventions, hence we propose to undertake a feasibility RCT first. The challenges include low engagement rates and attrition (non-use or fall-off in engagement with the intervention [17]), which limit their effectiveness, and high dropout rates (e.g. failure to complete follow-up questionnaires) seen in online trials [18]. There is also the potential for widening the digital divide, leading to health inequalities, as people from more deprived groups are less likely to have access to the internet and older people may have limitations in readiness and capacity to use online technologies [19, 20]. In light of these challenges, we feel that a feasibility study is required prior to applying for funding to carry out a Phase 3 RCT designed to test the hypothesis that supported access to an online alcohol intervention which mirrors treatment in community alcohol services is as effective, and more cost-effective, than traditional face to face treatments.

### Aims of the trial

The overall aim of this study is to determine the feasibility of conducting a RCT comparing supported access to a web-based Tier 2/3 treatment called Healthy Living for People who use Alcohol (HeLP-Alcohol) with usual care (face-to-face treatment in community alcohol services). The feasibility study will determine recruitment and retention rates and acceptability of the intervention; will test the online randomisation and data collection instruments at baseline and at 3 months; and will test methods to promote participant engagement with the intervention. We will also estimate health professional time involved in facilitating both arms of the trial. The outcomes of this feasibility study will inform a fully powered Phase 3 randomised controlled effectiveness trial and an economic evaluation.

## Methods

### Design

This is a randomised controlled feasibility trial.

### Setting

The London boroughs of Camden, Islington and Haringey have agreed to participate in the study, and we have recruited four community alcohol services to take part. Teams will be trained in good clinical practice (including trial paperwork, confidentiality, data management/clinical governance) and supported in recruiting and consenting participants.

### Population

We will recruit hazardous and harmful drinkers, identified using a validated screening tool such as AUDIT and referred from primary care, secondary care or self-referred, to community alcohol services.

- *Inclusion criteria*: patients are eligible to take part in the trial if they are aged 18 or over at time of screening; have a diagnosis of an alcohol use disorder using AUDIT criteria (score 8 or over); have a stable place of residence; provide informed consent for randomisation, treatment and follow-up; are willing and able to use a computer to access an online alcohol treatment programme.
- *Exclusion criteria*: patients are excluded from the trial if they have undergone treatment for substance use or primary drug dependence (excluding nicotine) in the previous 90 days; are already receiving help for an alcohol use disorder; have outstanding legal issues likely to lead to imprisonment; have severe physical dependency on alcohol (Leeds Dependence Questionnaire >20) or severe and complex co-existing physical or mental health problems, or are at risk of self-harm or suicide; are unable to consult in English without an interpreter; or if they are pregnant. If alcohol counsellors have concerns about a client regarding child protection issues or domestic violence, they will also be excluded. Patients without prior internet experience or without home access to the internet will not be excluded, but will be offered additional training as part of their facilitated access to the web-based alcohol treatment programme. All participants will be given information about local free or low-cost public internet access points, such as libraries, health centres and cluster rooms and local sources of support.

### Intervention

Supported access to HeLP-Alcohol, a modular, web-based alcohol treatment programme for people who drink at hazardous and harmful levels but who are not dependent on alcohol. HeLP-Alcohol is designed to be used over a 6-week period and has three evidence-based phases based on Motivational Interviewing, Computerised Cognitive Behavioural Therapy, and Relapse Prevention. Users can set up tailored text message or email prompts to help them achieve their drinking goals. Participants will also receive a weekly text message, email or phone call to encourage use of the website.

### Comparator

Treatment as usual (TAU) at participating community alcohol services.

### Recruitment

Clients will attend their assessment appointment with an alcohol counsellor as usual. If the alcohol counsellor considers the client meets the inclusion criteria for the trial (detailed above) they will discuss the trial with the client and provide written information and ask them to sign the consent form if they express interest in taking part. The client will be given a 24-hour “cooling off” period to decide whether or not to proceed.

### Data collection

**Fig. 1:**
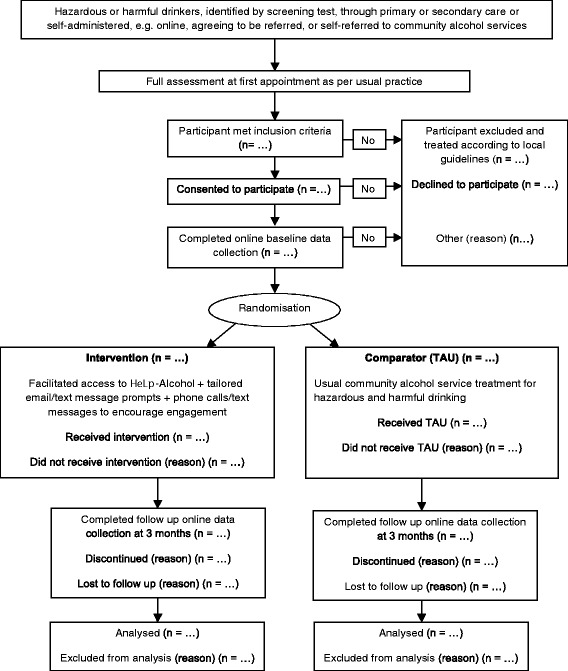
CONSORT participant flow diagram

If the client wishes to participate, they will be allocated a trial identification number linked to their email address and mobile phone number. All data will be collected anonymously online. All participants will be emailed a link to complete the baseline data collection questionnaires (see outcome measures, below). After they have completed the questionnaires, they will then be individually randomised by computer to the intervention or TAU. Those randomised to the intervention will be emailed a link to HeLP-Alcohol and those randomised to TAU will be directed back to their community alcohol service. All participants will be emailed another request for follow-up data collection 3 months after randomisation. For those participants who do not respond to the first email, there will be another two follow-up emails and then a phone call or text message request. See Fig. 1 for CONSORT participant flow diagram [21].

### Outcome measures

#### Primary outcomes

1. Recruitment as a percentage of eligible patients.
2. Retention measured by completeness of online data collection for each arm at baseline and at 3 months as a percentage of patients randomised.

#### Secondary outcomes

3. Demographic characteristics of participants recruited to each arm: age, sex, ethnic group, highest educational attainment and area deprivation (measured by Index of Multiple Deprivation) will be recorded at baseline, which will enable us to determine if there are inequalities in retention and use of the online intervention (for those randomised to this arm).
4. Outcome measures to be collected at baseline and at 3 months will include:
  - Unit consumption of alcohol per week (using TOT-AL, an online beverage-specific measure [22] which requires participants to enter the type and quantity of alcohol drinks consumed on each day of the past week)
  - Alcohol Use Disorders Identification Test (AUDIT) score [6], a method of screening for excessive drinking, developed by the World Health Organization (WHO) for use across a range of health settings
  - Current psychological global distress score (using CORE-10 [23] developed and validated as a non-proprietary measure of psychological distress)
  - Well-being scores (using EQ-5D [24])
  - Self-efficacy (using SCQ-8 [25]).
5. Outcome measures to be collected at 3 months:
  - Adherence to the intervention (for those randomised to this arm), measured through automated recording of numbers of log-ins and numbers of pages visited at each log-in
  - Other sources of support accessed during treatment (e.g. group therapy; horticulture; acupuncture; art therapy; other therapies)
  - Participant satisfaction with care (measured using the 8-item Client Satisfaction Questionnaire (CSQ-8), developed as a measure of satisfaction for mental health services, [26, 27] and used for assessing satisfaction with alcohol and other substance misuse programme) [28].

### Sample size

As this is a feasibility study a formal sample size calculation is not required, but we estimated the number of participants required as around 10 % of the number required for the Phase 3 trial [29]. The sample size calculation for the Phase 3 trial suggests we need to recruit 1665 participants. Given the participant population, a high level of attrition may be anticipated. We therefore aim to recruit 200 participants to the feasibility trial to inform the design and sample size of the Phase 3 RCT.

### Data handling and record keeping

Data from the website will be saved to a trial database which complies with the requirements of Good Clinical Practice and is maintained by the website developer. Data will be exported for analysis via UCL’s Identifiable Data Handling Solution (IDHS), which utilises a secure link to a secure drop box using ID numbers only and no identifying information. Participant contact details will be kept in a locked filing cabinet on university premises. Participant information at trial sites will also be kept securely according to local policies. The project has been registered with the UCL Data Protection Officer according to UCL Data Protection Policy.

### Statistical analysis

As this is a feasibility RCT, no hypothesis is to be tested. The aim of the trial is to determine estimates of recruitment and retention rates, to determine acceptability of the intervention and to provide parameters for the power calculation for a Phase 3 trial. Success criteria for recruitment and retention will be 84 and 75 %, respectively, as per the UKATT trial [30].

The analysis will be mainly descriptive. Descriptive statistics will be used to compare demographic characteristics of those completing each arm of the trial with those lost to follow-up. We will use multivariate logistic regression with robust confidence intervals (CI) to examine the impact of age, gender, ethnicity and deprivation level on these outcomes.

We will perform an analysis using regression of outcome on randomised group, adjusted for baseline value of outcome (for those outcomes measured at baseline) and other variables (age, gender, ethnicity and deprivation level) to compare the two arms using the following outcome measures at 3 months. Differences between the intervention and control groups for continuous variables with skewed distribution, e.g. alcohol consumption, will be log transformed, following a similar analysis to that by Wallace et al. [31]:

- Change in mean unit consumption of alcohol per week;
- Change in AUDIT score;
- Change in level of current psychological global distress score;
- Change in self-efficacy score;
- Acceptability of intervention using CSQ-8;
- Use of additional sources of support accessed during the trial period.

For the intervention group, we will examine adherence to the intervention, measured through automated recording of numbers of log-ins and numbers of pages visited at each log-in. We will conduct within-group analysis using linear regression to examine the impact of age, gender, ethnicity and deprivation on use of the intervention (number of log-ins, number of pages accessed, number of modules completed).

Missing data will be handled as follows:

- The primary analysis will be the mean difference in changes from baseline in all outcome variables between intervention and control groups at 3 months, using all available results but without imputation of missing data. Secondary analyses will include all randomised individuals by assuming that non-responders have no change in any missing outcome measures;
- For use of the intervention, data will be largely complete as automatically collected by the website.

### Trial status

We started recruiting patients to the trial in September 2014. Recruitment will run for 1 year, with data collection complete by end-December 2015.

## Discussion

Hazardous and harmful alcohol use is a growing problem in the UK. Face-to-face treatment is only accessed by around 10 % of those who could benefit, for several reasons, of which the most important are lack of provision, stigma and inconvenience. We have developed a psychologically informed web-based alcohol programme which may help fill this gap in access, but its effectiveness, cost-effectiveness and equality of access compared with usual treatment in community alcohol services need to be ascertained in a fully powered RCT. The results of this feasibility RCT will provide useful evidence for commissioners of alcohol services as previous studies of web-based treatments for hazardous and harmful alcohol use have been conducted mainly online, for example, comparing websites with and without interaction or feedback [31].

Conducting a Phase 3 RCT to compare a web-based alcohol treatment programme with face-to-face community alcohol treatment is likely to face challenges, hence the need for the feasibility study. Recruitment and retention rates in the feasibility RCT will give an indication of the acceptability of the trial to patients with problem alcohol use. Other methods such as qualitative interviews may give a better idea of acceptability or otherwise, both of the trial and the intervention, and we aim to undertake a separate qualitative study with participants and alcohol counsellors in due course. However, it may not be possible to get the views of people who decline to participate in the trial as they are likely to decline to take part in a qualitative study as well. It is also not possible to determine the characteristics of those who decline to take part as we are not able to collect their demographic data if they have not consented to be in the study. However, we will have access to summary data for all clients seen by a service as the services collect this data for their own evaluations, so this may give us some indication of the characteristics of those who decline to participate compared with those who consent.

It may be that potentially eligible clients do not wish to participate in a trial where there is a chance of being randomised to a website when they may have a preference for face-to-face counselling, or they may not have easy access to a computer, or feel confident using a computer. If they mention a reason for not participating when their alcohol counsellor discusses the trial with them, then we may pick this up from the qualitative interviews with counsellors.

Alcohol counsellors themselves may not find it acceptable to recruit people to a trial where they have a 50 % chance of being randomised to a website. The counsellors may only ask people who they think will be computer-literate, or may not ask those who they worry might disengage with the service if they are not seen face-to-face (selection bias). There may be other reasons why counsellors do not recruit potential recruits which may come out in the interviews, including time pressures and competing priorities.

We chose community alcohol services to as a recruitment setting because we expected that our target population would present to these services in sufficient numbers. However, as we are excluding hazardous or harmful drinkers who have co-morbidities or complex problems such as other substance misuse, eligible clients may not in fact present in sufficient numbers to recruit successfully from this setting.

As previously discussed, attrition from online programmes and online trials is a common problem [17]. We hope to overcome this using text message, email and telephone prompts as previous studies suggests such prompts can improve engagement [32]. We will also collect data on other sources of help accessed during the study, participants’ satisfaction with the intervention and estimates of health professional time for each arm, which will inform the main trial.

The proposed feasibility study is therefore important in order to identify these challenges prior to designing the Phase 3 trial, to test online data collection, randomisation methods and the prompts to encourage continued use of the intervention. Data from this study will also be used to refine the sample size calculation for the Phase 3 RCT. Our hypothesis is that supported access to HeLP-Alcohol is of equal effectiveness as TAU. The RCT will be designed to have 90 % power to detect (at a 5 % significance level) a difference in mean weekly alcohol consumption of 15 % between intervention and TAU groups (if such a difference occurs). The assumptions are (i) standard deviation of alcohol consumption at follow-up is equal to 85 % of the mean and (ii) a correlation between baseline and follow-up alcohol consumption of 0.40, as found in a previous RCT of an online alcohol intervention [30]; (iii) a follow-up rate of 75 % at 12 months, based on the experience of UKATT [33]; (iv) an intra-cluster correlation of 0.02 for the effect of alcohol counsellors, also based on the UKATT trial. The standard error of the mean difference in alcohol consumption will be about 5 %, so an estimated zero difference would have a 95 % confidence interval of −10 to +10 %. The definitive trial therefore requires 1665 participants.

If the definitive study shows that a web-based alcohol treatment programme is as effective as face to face treatment, then implementation of such a programme could increase patient choice, improve access to alcohol treatment for hazardous and harmful drinkers and lead to cost savings for treatment services and more widely for the NHS in treating alcohol-related health conditions, particularly for people who are reluctant or unable to attend face-to-face services.

## Abbreviations

A&E: accident and emergency
AUDIT: Alcohol Use Disorders Identification Test
AUDIT-C: Alcohol Use Disorders Identification Test, Consumption
CSQ-8: Client Satisfaction Questionnaire
CORE-10: Clinical Outcomes in Routine Evaluation (10 item)
EQ-5D: Euroqol 5-dimensions
FAST: Fast Alcohol Screening Test
HeLP-Alcohol: Healthy Living for People who use Alcohol
IBA: identification and brief advice
NHS: National Health Service
NRES: National Research Ethics Committee
SCQ-8: Situational Confidence Questionnaire (8 item)
SBI: screening and brief intervention
TAU: treatment as usual
TOT-AL: TOTal past week ALcohol consumption
UK: United Kingdom
WHO: World Health Organization

## References

[1] Rehm J, Mathers C, Popova S, Thavorncharoensap M, Teerawattananon Y, Patra J (2009). Global burden of disease and injury and economic cost attributable to alcohol use and alcohol-use disorders. Lancet.

[2] NICE, National Institute of Clinical Excellence (2010). Alcohol-use disorders: preventing the development of hazardous and harmful drinking. NICE Public Health Guidance 24. London.

[3] HM Government (2012). The government’s alcohol strategy.

[4] Drummond C, Oyefoso A, Phillips T, Cheeta S, Deluca P, Perryman K (2005). Alcohol Needs Assessment Research Project (ANARP). The 2004 National Alcohol Needs Assessment for England.

[5] Department of Health/National Treatment Agency for Substance Misuse (2006). Models of care for alcohol misusers (MoCAM).

[6] Bohn MJ, Babor TF, Kranzler HR (1995). The Alcohol Use Disorders Identification Test (AUDIT): validation of a screening instrument for use in medical settings. J Stud Alcohol.

[7] Bradley KA, DeBenedetti AF, Volk RJ, Williams EC, Frank D, Kivlahan DR (2007). AUDIT-C as a brief screen for alcohol misuse in primary care. Alcohol Clin Exp Res.

[8] Hodgson R, Alwyn T, John B, Thom B, Smith A (2002). The FAST alcohol screening test. Alcohol Alcohol.

[9] NICE (2010). Alcohol-use disorders: preventing the development of hazardous and harmful drinking. NICE Public Health Guidance 24.

[10] Kaner EFS, Dickinson HO, Beyer F, Pienaar E, Schlesinger C, Campbell F (2009). The effectiveness of brief alcohol interventions in primary care settings: a systematic review. Drug Alcohol Rev.

[11] Purshouse RC, Brennan A, Rafia R, Latimer NR, Archer RJ, Angus CR (2013). Modelling the cost-effectiveness of alcohol screening and brief interventions in primary care in England. vol 2.

[12] Commander M, Odell S, Williams K, Sashidharan S, Surtees P (1999). Pathways to care for alcohol use disorders. J Public Health.

[13] Drummond DC (2004). An alcohol strategy for England: the good, the bad and the ugly vol 5.

[14] Office for National Statistics (2014). Internet access – households and individuals.

[15] Khadjesari Z, Murray E, Hewitt C, Hartley S, Godfrey C (2011). Can stand-alone computer-based interventions reduce alcohol consumption? A systematic review. Addiction.

[16] Murray E, Linke S, Harwood E, Conroy S, Stevenson F, Godfrey C (2012). Widening access to treatment for alcohol misuse: description and formative evaluation of an innovative web-based service in one primary care trust. Alcohol Alcohol.

[17] Murray E, Khadjesari Z, White IR, Kalaitzaki E, Godfrey C, McCambridge J (2009). Methodological challenges in online trials. J Med Internet Res.

[18] Eysenbach G (2005). The law of attrition. J Med Internet Res.

[19] Ralston JD, Hirsch IB, Hoath J, Mullen M, Cheadle A, Goldberg HI (2009). Web-based collaborative care for type 2 diabetes: a pilot randomized trial. Diabetes Care.

[20] Viswanath K, Kreuter MW (2007). Health disparities, communication inequalities, and eHealth. Am J Prev Med.

[21] Moher D, Schulz KF, Altman DG (2001). The CONSORT statement: revised recommendations for improving the quality of reports of parallel group randomized trials. BMC Med Res Methodol.

[22] Khadjesari Z, Murray E, Kalaitzaki E, White IR, McCambridge J, Godfrey C (2009). Test-retest reliability of an online measure of past week alcohol consumption (the TOT-AL), and comparison with face-to-face interview. Addict Behav.

[23] Barkham M, Bewick B, Mullin T, Gilbody S, Connell J, Cahill J et al. The CORE-10: A short measure of psychological distress for routine use in the psychological therapies. Counsell Psychother Res. 2013;13(1):pp. doi:10.1080/14733145.2012.729069.

[24] The EuroQol Group. A new facility for the measurement of health-related quality of life. Health Policy 1990;16(3):199-20. doi:10.1016/0168-8510(90)90421-9. 10.1016/0168-8510(90)90421-9 10109801

[25] Breslin FC, Sobell LC, Sobell MB, Agrawal S (2000). A comparison of a brief and long version of the Situational Confidence Questionnaire. Behav Res Ther.

[26] Larsen DL, Attkisson CC, Hargreaves WA, Nguyen TD (1979). Assessment of client/patient satisfaction: development of a general scale. Eval Program Plann.

[27] Attkisson CC, Zwick R (1982). The client satisfaction questionnaire. Psychometric properties and correlations with service utilization and psychotherapy outcome. Eval Program Plann.

[28] Dearing RL, Barrick C, Dermen KH, Walitzer KS (2005). Indicators of client engagement: influences on alcohol treatment satisfaction and outcomes. Psychol Addict Behav.

[29] Cocks K, Torgerson DJ (2013). Sample size calculations for pilot randomized trials: a confidence interval approach. J Clin Epidemiol.

[30] Kaner E, Bland M, Cassidy P, Coulton S, Deluca P, Drummond C (2009). Screening and brief interventions for hazardous and harmful alcohol use in primary care: a cluster randomised controlled trial protocol. BMC Public Health.

[31] Wallace P, Murray E, McCambridge J, Khadjesari Z, White IR, Thompson SG (2011). On-line randomized controlled trial of an internet based psychologically enhanced intervention for people with hazardous alcohol consumption. PLoS One.

[32] Alkhaldi G, Hamilton FL, Lau R, Webster R, Michie S, Murray E (2015). The effectiveness of technology-based strategies to promote engagement with digital interventions: a systematic review protocol. JMIR Res Protoc.

[33] Effectiveness of treatment for alcohol problems: findings of the randomised UK alcohol treatment trial (UKATT). BMJ. 2005;331(7516):541. 10.1136/bmj.331.7516.541 PMC1200586 16150764

